# Assessment of thiamine status and its association with clinical parameters in patients undergoing maintenance hemodialysis

**DOI:** 10.3389/fnut.2025.1563768

**Published:** 2025-05-14

**Authors:** Bo Yang, Naiying Lan, Fanzhou Zeng, Qing Shao, Dan Ye, Hao Wang, Cheng Xue, Nanmei Liu

**Affiliations:** ^1^Department of Nephrology, Naval Medical Center of PLA, Naval Medical University, Shanghai, China; ^2^Division of Nephrology, Changzheng Hospital, Naval Medical University, Shanghai, China

**Keywords:** thiamine, hemodialysis, anemia, iron metabolism, cross-sectional study

## Abstract

**Objective:**

Thiamine deficiency is a common complication in end-stage renal disease (ESRD) patients receiving maintenance hemodialysis (HD). The purpose of this cross-sectional study was to assess the prevalence of thiamine deficiency in HD patients and its association with clinical parameters.

**Methods:**

This was a single-center cross-sectional study that included 113 maintenance HD patients from our hospital. Thiamine status was evaluated by high-performance liquid chromatography on whole blood samples. We evaluated the association between blood thiamine concentration and other clinical parameters, including markers of iron metabolism and cardiac function.

**Results:**

The prevalence of thiamine deficiency was 11.5%. Univariate analysis revealed a significant positive correlation between thiamine levels and iron metabolism markers, including hemoglobin level (Rho = 0.257, *p* = 0.006), transferrin saturation (Rho = 0.244, *p* = 0.009), and serum iron (Rho = 0.213, *p* = 0.025). A multivariate regression analysis confirmed that thiamine levels were independently associated with hemoglobin levels (beta coefficients = 0.25, *p* = 0.012).

**Conclusion:**

These findings suggest an association between lower thiamine levels and anemia in HD patients. Further research is needed to elucidate the underlying mechanisms and evaluate the efficacy of thiamine supplementation in improving anemia and other clinical outcomes in this population.

## 1 Introduction

End-stage renal disease (ESRD) is a chronic, life-threatening condition that requires renal replacement therapy, primarily in the form of hemodialysis (HD) (1). While HD is essential for survival, it is associated with a number of complications, including malnutrition, metabolic disturbances, and an increased risk of cardiovascular disease (2).

Thiamine, a water-soluble B vitamin, is essential for several metabolic processes, including carbohydrate metabolism, energy production, and nerve function (3). In individuals with ESRD, thiamine deficiency is a common occurrence (4), exacerbated by factors such as reduced dietary intake, impaired absorption, increased catabolism, and dialyzer clearance (5).

Asian dietary patterns, which are often characterized by high carbohydrate and low protein intake, can contribute to thiamine deficiency in HD patients. This is because thiamine is primarily found in whole grains, legumes, and meat, which may be less common in traditional Asian diets (6).

Given the potential impact of thiamine deficiency on the health and prognosis of HD patients, the KDOQI clinical practice guideline for nutrition in CKD recommended treatment with multivitamins, including thiamine, in HD patients with inadequate dietary intake (7). However, the quality of the evidence was insufficient to support the statement’s classification as ‘opinion’. It is essential to assess thiamine status in the population with maintenance HD. The primary objective of this cross-sectional study is to determine the prevalence of thiamine deficiency among maintenance HD patients at our center. Additionally, we aim to investigate the association between blood thiamine levels and key prognostic factors such as age, diabetes mellitus, and cardiovascular disease.

Understanding the prevalence and determinants of thiamine deficiency in HD patients allows us to identify individuals at risk and implement appropriate interventions to optimize their nutritional status and clinical outcomes.

## 2 Methods

The research has been registered in chictr.org.cn (ChiCTR2400093762).

### 2.1 Study participants

This was an exploratory, single-center investigation. A formal sample size calculation was not performed *a priori*; all eligible and consented patients receiving maintenance HD at the center during the study period were invited to participate. Inclusion criteria of the current study were (1) patients aged 18 years or older; and (2) patients receiving maintenance HD three times/week at Naval Medical Center of the PLA. Exclusion criteria included (1): patients who had combined therapy with peritoneal dialysis and HD, (2) patients with any unstable clinical conditions, (3) patients who were unable to actively ingest food orally, (4) patients who had undergone general anesthesia surgery within the past week, and (5) patients who refused to participate in this study. Data on thiamine supplement usage were collected; only one patient included in the study was taking thiamine supplements. Informed consent was obtained from each subject enrolled in this study. The institutional review committee of the Naval Medical Center of the PLA approved this research. The study was conducted in accordance with local legislation and institutional requirements.

### 2.2 Data collection

We collected data on age, sex, body mass index, primary cause of renal failure, dialysis vintage, complications, and comorbidities such as cardiovascular diseases, diabetes, and stroke. Cardiac ultrasound parameters including LVEF, sPAP, and E/e’. Major lab test results include iron metabolism biomarkers, BNP, Pro-BNP, albumin, c-reactive protein, blood urine nitrogen, pre-albumin, total cholesterol, hemoglobin, calcium (including albumin-corrected calcium), phosphorus, and parathyroid hormone. The routine laboratory tests were performed using standard automated methods in the hospital’s certified clinical laboratory. Thiamine status was evaluated by high-performance liquid chromatography measurement of thiamine in whole blood, performed by a certified external laboratory (KingMed Diagnostics, Inc., Guangzhou, China) using an Agilent 1,290 Infinity LC System. A single blood sample was performed before dialysis.

### 2.3 Statistical analyses

Continuous variables were expressed as means ± standard deviation (SD) and evaluated with a *t*-test when data had a normal distribution. Otherwise, continuous variables were expressed as medians and interquartile ranges and evaluated using Mann–Whitney U-test. Categorical variables were expressed as percentages and analyzed with the chi-square test. Depending on the distribution of thiamine levels, Pearson’s or Spearman’s correlation was used to determine correlations between blood thiamine concentration and multiple parameters. Furthermore, a multivariate linear regression analysis was conducted to investigate the clinical significance of lower blood thiamine levels. Statistical analysis was conducted using Statistics Software for MacOS, version 30.0.0.0 (SPSS Inc., Chicago, IL, USA), with significant differences determined at a *p*-value < 0.05.

## 3 Results

### 3.1 Baseline characteristics

**Figure 1:**
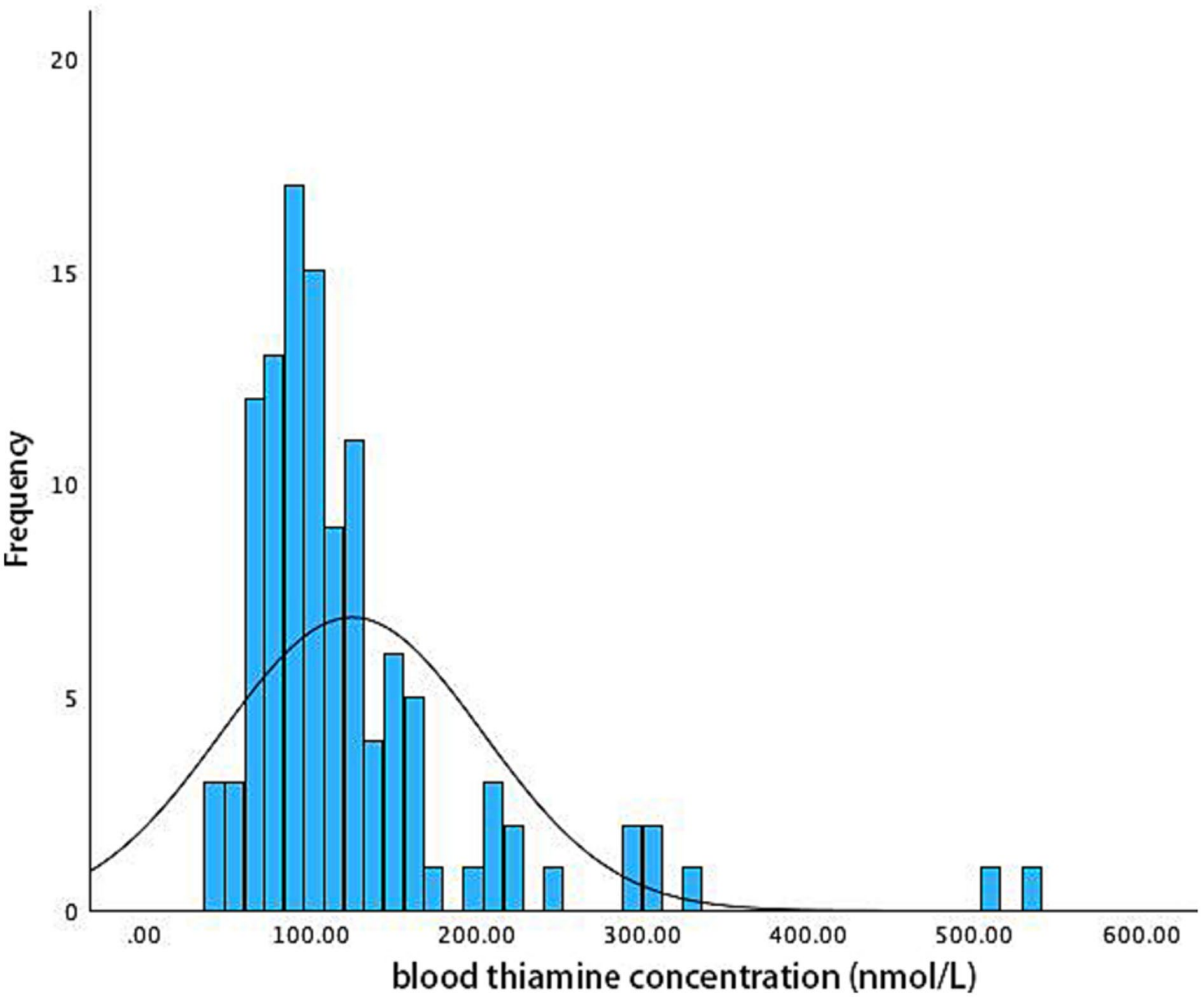
Histogram shows the distribution of blood thiamine levels in the study population. In 13 (11.5%), blood thiamine concentration was below the lower limit of the reference range (< 70 nmol/L).

There are 113 subjects enrolled in the current study. The baseline characteristics of the study participants are shown in Table 1. The distribution of blood thiamine levels in the enrolled patients is shown in Figure 1. The data exhibit a non-normal distribution. The median value is 104.3 nmol/L, with an interquartile range (IQR) of 81.85 nmol/L to 138.4 nmol/L, indicating that the data are skew. According to the lower limit of normal blood thiamine level (70 nmol/L), as defined by the reference range of the testing laboratory, 13 (11.5%) patients were defined as thiamine deficiency.

**Table 1:** Baseline characteristics of study subjects.

Characteristic	Value (*n* = 113)
Age (years)	66.00 [55.25, 70.75]
Male gender [*n* (%)]	50 (53.1%)
Dialysis vintage (years)	5.0 [2, 10]
BMI (kg/m^2^)	22.20 (3.85)
Hypertension	112 (99.1%)
Cardiovascular diseases	109 (96.46)
Type 2 diabetes	37 (32.7%)
Blood thiamine level (nmol/L)	104.3 [81.85, 138.4]
Primary cause of renal failure	
Glomerular nephritis	43 (38.1%)
Diabetic nephropathy	30 (26.5%)
Hypertensive nephrosclerosis	10 (8.8%)
Polycystic kidney disease	8 (7.1%)
Kidney cancer	5 (4.4%)
Interstitial nephritis	3 (2.7%)
Graft failure	3 (2.7%)
Others	11 (9.7%)
Lab tests	
BNP (pg/mL)	192.7 [87.05, 591.3]
Pro-BNP (pg/mL)	5436.5 [2502.5, 19799.5]
Alb (g/L)	40.9 [37.9, 42.7]
Pre-alb (mg/L)	283.55 [226.5, 343.77]
CRP (mg/L)	4.53 [1.05, 10.64]
BUN (mmol/L)	21.73 [18.12, 26.65]
TC (mmol/L)	3.23 [2.56, 3.85]
Hb (g/dL)	104 [97, 116]
PTH (pg/mL)	191.15 [76.82, 377.63]
Ca (mmol/L)	2.35 [2.18, 2.51]
CCA (mmol/L)	2.33 [2.12, 2.58]
P (mmol/L)	1.74 [1.35, 2.21]
TSAT (%)	0.19 [0.12, 0.28]
Ferritin (ng/mL)	33.89 [20.09, 73.19]
Serum iron (ug/dL)	51.6 [36.8, 80.5]
Transferrin (g/L)	2.39 [2.1, 2.71]
UIBC (ug/dL)	248.3 [188.1, 299.9]
ESA use	85 (75.2%)
Iron supplements	23 (20.4%)
HIF-PH inhibitors use	59 (52.2%)
Cardiac ultrasound parameters	
LVEF	0.61 [0.58, 0.65]
sPAP (mmHg)	36 [30, 42]
E/e’	12.3 [8.9, 16.2]

Continuous variables are expressed as median [interquartile range]. BMI, body mass index; BNP, B-type natriuretic peptide; alb, albumin, pre-alb, pre-albumin; CRP, c-reactive protein; BUN, blood urine nitrogen; TC, total cholesterol; Hb, hemoglobin, PTH, parathyroid hormone; CCA, calcium corrected for albumin; TSAT, transferrin saturation; UIBC, unsaturated iron binding capacity; ESA, erythropoiesis-stimulating agent (specific human erythropoietin injection (Shenyang Sunshine Pharmaceuticals CO., Ltd) in our center); HIF-PH, hypoxia-inducible factor prolyl hydroxylase; LVEF, left ventricular rejection fraction; sPAP, systolic pulmonary artery pressure.

### 3.2 Univariate correlations between blood thiamine level and other variables

To investigate the clinical significance of blood thiamine concentration, we performed univariate analysis using Spearman’s correlation of the association between blood thiamine level and other clinical and laboratory parameters (Table 2). All analyses in this section, including the multivariate analysis, were conducted using data from all 113 participants. Thiamine levels did not correlate with age, sex, body mass index, primary cause of renal failure, dialysis vintage, complications, and comorbidities. In light of thiamine’s critical role in maintaining cardiac function, this study explored the association between thiamine levels and various cardiac function assessment indicators. Nevertheless, our results indicated no significant correlation between thiamine levels and BNP, Pro-BNP, echocardiographic parameters (LVEF, sPAP, E/e’), or H_2_FPEF score (8, 9), implying that our study cohort found no association between thiamine and the occurrence of heart failure. Of note, thiamine levels correlated positively with hemoglobin (Rho = 0.257, *p* = 0.006), transferrin saturation (Rho = 0.244, *p* = 0.009), and serum iron (Rho = 0.213, *p* = 0.025), but negatively with unsaturated iron-binding capacity (Rho = −0.218, *p* = 0.021). In CKD-MBD lab testing, thiamine levels were positively correlated with phosphorus (Rho = 0.273, *p* = 0.003) but not with PTH or calcium (including calcium-corrected for albumin).

**Table 2:** Univariate correlations between blood thiamine concentration and clinical and laboratory parameters.

Variable	Spearman Rho	*p* value
age (years)	−0.031	0.744
dialysis vintage (years)	0.122	0.212
LVEF (%)	0.017	0.865
BMI (kg/m2)	0.159	0.140
sPAP (mmHg)	−0.076	0.435
E/e’	−0.067	0.508
BNP (pg/mL)	−0.007	0.959
Pro-BNP (pg/mL)	−0.094	0.329
H_2_FPEF Score	−0.005	0.955
alb (g/L)	0.118	0.217
CRP (mg/L)	−0.031	0.746
BUN (mmol/L)	0.187	0.052
pre-alb (mg/L)	0.105	0.276
TC (mmol/L)	0.016	0.868
Hb (g/dL)	0.257**	0.006
PTH (pg/mL)	0.162	0.091
Ca (mmol/L)	0.090	0.350
CCA (mmol/L)	−0.077	0.419
P (mmol/L)	0.273**	0.004
TSAT (%)	0.244**	0.010
ferritin (ng/mL)	0.088	0.405
serum iron (ug/dL)	0.213*	0.025
transferrin (g/L)	−0.084	0.379
UIBC (ug/dL)	−0.218*	0.021
ESA use	−0.132	0.167
iron supplements	0.009	0.928
HIF-PH inhibitors use	−0.046	0.630

LVEF, left ventricular rejection fraction; BMI, body mass index; sPAP, systolic pulmonary artery pressure; BNP, B-type natriuretic peptide; H_2_FPEF, diagnostic algorithm for heart failure with preserved ejection fraction; alb, albumin; CRP, c-reactive protein; BUN, blood urine nitrogen; TC, total cholesterol; Hb, hemoglobin; PTH, parathyroid hormone; Ca, calcium; CCA, calcium corrected for albumin; P, phosphorus; TSAT, transferrin saturation; UIBC, unsaturated iron binding capacity; ESA, erythropoiesis-stimulating agent; HIF-PH, hypoxia-inducible factor prolyl hydroxylase. ^*^p < 0.05. ^**^p < 0.01.

### 3.3 Multivariate regression analysis of factors influencing hemoglobin level

Univariate correlation analysis revealed a significant correlation between thiamine levels and a panel of iron metabolism parameters, including hemoglobin level. To determine factors independently related to lower hemoglobin levels in the study population, we further performed an enter method multivariate regression analysis that included quartiling thiamine levels, albumin, CRP, serum iron, TSAT, UIBC, ESA use, iron supplementation, and hypoxia-inducible factor prolyl hydroxylase inhibitors use (Table 3). We found that only quartiling thiamine levels were independently correlated with blood hemoglobin levels (beta coefficients = 0.25, *p* = 0.012).

**Table 3:** Enter multivariate regression analysis influencing hemoglobin level.

Variable	Unstandardized coefficient B	Std. error	Standardized coefficient beta	*t*	*p*-value
TSAT	−1.412	1.571	−0.088	−0.898	0.371
Serum iron	−0.064	0.056	−0.137	−1.154	0.251
UIBC	0.003	0.03	0.013	0.113	0.91
ESA use	−4.972	4.898	−0.102	−1.015	0.312
Iron supplements	0.925	5.323	0.018	0.174	0.862
HIF-PH inhibitors use	−1.383	4.173	−0.034	−0.332	0.741
Quartiling thiamine levels	4.58	1.793	0.25*	2.554	0.012
Alb	0.736	0.392	0.188	1.875	0.064
CRP	−0.079	0.079	−0.102	−0.992	0.324

TSAT, transferrin saturation; UIBC, unsaturated iron binding capacity; ESA, erythropoiesis-stimulating agent; HIF-PH, hypoxia-inducible factor prolyl hydroxylase; alb, albumin; CRP, c-reactive protein. ^*^p < 0.05.

## 4 Discussion

This cross-sectional study aimed to assess the prevalence of thiamine deficiency among maintenance HD patients and its association with various clinical parameters. Our findings indicate that a significant proportion of HD patients exhibit thiamine deficiency. This finding aligns with the results of earlier research (4, 10–12). However, Ubutata et al. (12) found no association between thiamine levels and nutritional markers such as albumin or hemoglobin.

While thiamine levels did not correlate with traditional cardiovascular risk factors or cardiac function parameters, there was a positive association between thiamine levels and iron metabolism markers, particularly hemoglobin. The use of multivariate analysis compared our work with Ubukata et al. paper (12). Our analysis suggests that the association between thiamine levels and hemoglobin is independent of several other factors known to influence anemia in HD patients, which was not found in the Ubukata et al. study.

Thiamine deficiency is a common complication in HD patients, potentially contributing to various metabolic derangements and adverse clinical outcomes (13). The mechanisms underlying the association between thiamine and iron metabolism are complex and require further investigation. However, it is possible that thiamine may play a role in iron absorption or utilization, or that it indirectly influences iron metabolism through its impact on other metabolic pathways (14).

While the exact mechanisms underlying the observed association between thiamine deficiency and anemia are not fully understood, several plausible explanations can be considered: (1) oxidative stress: Thiamine, an antioxidant, can protect cells from oxidative damage. In thiamine deficiency, increased oxidative stress can damage red blood cells, resulting in hemolysis and anemia (15, 16). (2) inflammation: Thiamine deficiency can lead to increased inflammation, as evidenced by elevated levels of inflammatory markers (17). Chronic inflammation can impair iron absorption and utilization, resulting in anemia (17, 18). (3) Neurological dysfunction: Thiamine is essential for the synthesis of neurotransmitters, including acetylcholine (3). Dysfunction of the nervous system, particularly the autonomic nervous system, can affect the regulation of hematopoiesis and iron metabolism (19, 20).

Our study highlights the importance of assessing thiamine status in HD patients and considering potential implications of low thiamine levels, particularly in those with concurrent anemia or evidence of impaired iron metabolism.

Several limitations should be acknowledged. First, this was a cross-sectional study, which limited our ability to establish causal relationships. Longitudinal studies are needed to further investigate the temporal relationship between thiamine levels and clinical outcomes. Second, the sample size was relatively small, which may have limited the statistical power to detect significant associations. Third, we did not include dietary intake or specific ESA dosages, which are potential confounding factors that may influence thiamine status and/or hemoglobin levels.

Future longitudinal studies are needed to investigate the long-term impact of thiamine supplementation on clinical outcomes in HD patients. In addition, to elucidate the underlying mechanisms linking thiamine to iron metabolism and other metabolic pathways, mechanistic studies are also needed. By addressing these limitations and pursuing future research, we can gain a deeper understanding of the role of thiamine in HD patients and develop effective strategies to optimize their nutritional status and clinical outcomes.

In conclusion, our study found a significant prevalence of thiamine deficiency among maintenance HD patients and highlights the potential independent association between thiamine levels and iron metabolism. Further research is warranted to confirm these findings and to investigate the underlying mechanisms and clinical implications of thiamine deficiency in this population.

## Data Availability

The original contributions presented in the study are included in the article/supplementary material, further inquiries can be directed to the corresponding authors.
